# Acute microbiologically negative hypoxic interstitial pneumonia on HAART: Immune Reconstitution Inflammatory Syndrome unmasking Pneumocystis Jiroveci infection with an atypical presentation

**Published:** 2012-06-18

**Authors:** S Sovaila, A de Raigniac, C Picard, O Taulera, C Lascoux-Combe, D Sereni, A Bourgarit

**Affiliations:** * Department of Internal Medicine, Saint-Louis Hospital, AP-HP, Paris Denis-Diderot University, Paris, France; ** Department of Respiratory Medicine, Saint-Louis Hospital, AP-HP, Paris Denis-Diderot University, Paris, France

**Keywords:** immune restoration inflammatory syndrome (IRIS), Pneumocystis jiroveci, HIV, unmasking, HAART

## Abstract

Highly active antiretroviral therapy for AIDS sometimes engenders inflammatory manifestations resulting from an inappropriate and unbalanced immune-system restoration, called Immune Reconstitution inflammatory Syndrome, which, in turn, can unmask a subclinical infection/pathology. Despite our patient’s evident syndrome, the atypical clinical, microbiologic and radiologic feature of Pneumocystis pneumonia made its diagnosis difficult.

## Introduction

Pneumocystis jiroveci pneumonia (PJP) is a frequent opportunistic infection occurring in moderately-severely immunodeficient HIV-infected patients before starting Highly Active Antiviral Therapy (HAART). Classical clinical and radiological manifestations include insidious general and respiratory symptoms, associated with multicystic alveolar–interstitial pneumonia.

Since the advent of HAART, inappropriate and, probably, unbalanced restoration of immune system functions has resulted in exaggerated inflammatory clinical manifestations characterizing the immune reconstitution inflammatory syndrome (IRIS). Two main IRIS types are described [**1**]: clinical deterioration of a known and treated condition (opportunistic infection, neoplasia) define paradoxical IRIS [**2**]; whereas the appearance of new symptoms leading to detection of a subclinical infection is an unmasking-IRIS [**3,4**]. This latter diagnosis may be particularly difficult to make because clinical symptoms differ from the classical infection-associated manifestations.

The present report describes an unmasking-IRIS–associated PJP with unusual and misleading clinical manifestations.

## Case report

A 34-year-old HIV-1-infected woman was admitted for dyspnea and acute bilateral pneumonia. HIV-1 infection had been diagnosed 8 years earlier during pregnancy, when she received only mother-to-child antiviral infection prevention. When her virus load (VL) reached 100000 copies/ml and CD4-cell count fell to 270/mm3 HAART with efavirenz, tenofovir and emcitrabine was started on 16 July 2008. Eight days later, she complained of fever and a persistent cough. Because no clinical improvement was obtained after 5 days of self-administered amoxicillin–clavulanate and ofloxacin, she was admitted; her chest X-ray showed bilateral interstitial pneumonia. Her diagnostic work-up found polypnea, cough and fever at 41°C, and a biologically severe inflammatory syndrome (C-reactive protein: 190 g/dL) without eosinophilia. Arterial blood-gas determination showed severe hypoxemia PO2 45.6 mm Hg. The thoracic computed tomography (CT) scan revealed multifocal areas of branched centrilobular nodules and patchy ground-glass opacities with few lobular reticulates (Fi), without cavitation, pleural involvement or mediastinal lymphadenopathy. Bronchoalveolar lavage (BAL) cytology showed 950,000 cells/ml: 33% macrophages, 16% neutrophils, 8% eosinophils, no mast cells and 43% lymphocytes, with a CD4/CD8 ratio of 0.6. Direct BAL microscopy was negative for Pneumocystis jiroveci cysts, Mycobacterium species, Candida and viral inclusions. Histological examination of bronchial biopsies showed aspecific chronic inflammatory infiltrates. At this time, her HIV-1 VL was 5,322 copies/ml and CD4 T-lymphocyte count had reached 510/mm3 (25%).

**Fig. 1:**
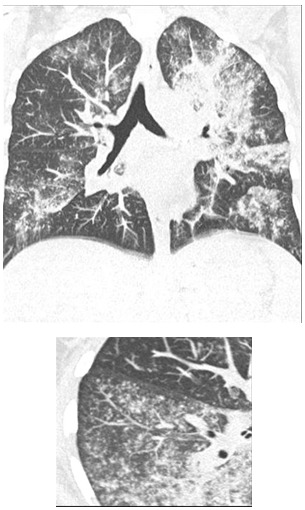
Volumetric reconstruction of the Thoracic computed tomography-scan at admission, showing multifocal areas of branched centrilobular nodules and patchy ground-glass opacities with few lobular reticulates (left). At right a single 5-mm thick slice of the upper right lobe.

Because of the worsening of the pulmonary function, the patient was transferred to the ICU and improved rapidly, when HAART was interrupted, under cotrimoxazole and steroids (1mg/kg/day) initiated for suspected IRIS, unmasking of PJP, which was subsequently confirmed, after the elimination of other possible diagnoses, by both BAL positive Pneumocystis-DNA polymerase chain reaction amplification and careful microscopic reanalysis showing a few parasitic cysts.

## Discussion

IRIS pathogenesis is reportedly linked to an acute and uncontrolled recovery of antigen-specific immune responses [**5**] and is associated with a broad nonspecific inflammatory syndrome. These immunologic and inflammatory pathogenetic pathways can explain why IRIS-associated clinical symptoms may differ from usual infection-related manifestations. Paradoxical IRIS associated with already diagnosed opportunistic infections is a well-described entity frequently arising in patients receiving effective HAART mostly reported during Mycobacterium tuberculosis and cryptococcal infections. PJP-associated paradoxical IRIS [**6**] is characterized by an acute worsening of fever, with severe respiratory failure and diffuse alveolar opacities. These well-described events have become rare because steroids are often prescribed and HAART delayed once PJP is diagnosed [**7**]. Unmasking-IRIS, recently separated from the former [**1,8**], is more difficult to diagnose and requires more urgent adaptation of therapy, because the underlying pathogen is not yet known or treated. To our knowledge, only a few cases of PJP-associated unmasking-IRIS, has been reported [**9,10**]. Our patient’s underlying PJP was especially difficult to discern because of several atypical clinical features. However, the rapid response obtained after stopping of HAART and starting specific antimicrobial therapy and high-dose steroids achieved a favorable clinical outcome. PJP-associated unmasking-IRIS was diagnosed in 2-steps. In light of the symptom kinetics soon after HAART initiation and concomitant with its good virologic and immunologic efficacy, the IRIS diagnosis was easily made, it was the search for and identification of the underlying opportunistic infection that posed the problem. Indeed, this patient had several atypical clinical and radiologic characteristics. The patient’s CD4 T-cell count was higher than usual in the presence of this opportunistic infection and could be explained by a qualitative rather than quantitative immunodeficiency [**11**]. Secondly, BAL fluid findings were atypical: in classical HIV-associated PJP, BAL usually contains numerous parasitic cysts which make the diagnosis possible on direct sputum examination [**12,13**]. In the PJP-associated paradoxical-IRIS cases described in the literature, BAL were mostly negative for Pneumocystis jiroveci cysts and diagnosis required recourse to molecular biology or thoracic biopsy. That situation is consistent with IRIS at the time of an already treated PJP. In unmasking-IRIS, like our patient’s, the absence of cysts cannot be explained by anti-parasitic drug efficacy. The small number of cysts and the appearance of mixed alveolitis, which is an atypical inflammatory response of the affected tissue, might suggest that the respiratory symptoms are secondary to an inappropriate immune-system reaction, rather than to the infection itself [**8**]. A more careful reexamination of the BAL provided a retrospective confirmation of our patient’s diagnosis. From a radiologic perspective, 9 out of 10 times, in PJP, and PJP-associated paradoxical IRIS, thoracic CT scans revealed exudative alveolitis resembling bilateral ground-glass attenuation [**13**]. The presence of multiple branched micronodules, as in our patient’s case, is not typical of PJP and usually suggests the presence of airway involvement, like that seen in infectious bronchiolitis or mycobacterial infection. Nodules have been described in PJP but are usually not the predominant sign [**14**]. Only Chen et al. reported such unusual PJP-associated unmasking-IRIS with granulomatous histologic lesions but their patient had PJP and Mycobacterium avium co-infections [**15**]. Thus, the radiologic appearance of PJP-associated unmasking-IRIS could differ from classic PJP or the generally described PJP-associated paradoxical-IRIS images, which may reflect to a specific pathogenetic process. Because infection-associated IRIS symptoms coincide with the burst of the acute reconstitution of anti-infectious immunity [**5**], clinical symptoms might be more attributable to the inflammatory immune responses to some exogenous antigens, than to the virulence of the infectious agent. In our opinion, atypical clinical symptoms arising after HAART introduction should evoke unmasking-IRIS and require concentrated efforts to identify the underlying pathogen, independently of the classical epidemiologic, clinical, radiologic or microbiologic descriptions. **Acknowledgements:** Special acknowledgment to Mrs. Janet Jacobson for her editing of the manuscript. No conflicts of interest
